# Synergic Interaction of Rifaximin and Mutaflor (*Escherichia coli *Nissle 1917) in the Treatment of Acetic Acid-Induced Colitis in Rats

**DOI:** 10.1155/2016/3126280

**Published:** 2016-06-28

**Authors:** Artur Dembiński, Zygmunt Warzecha, Piotr Ceranowicz, Marcin Dembiński, Jakub Cieszkowski, Tomasz Gosiewski, Małgorzata Bulanda, Beata Kuśnierz-Cabala, Krystyna Gałązka, Peter Christopher Konturek

**Affiliations:** ^1^Department of Physiology, Faculty of Medicine, Jagiellonian University Medical College, 16 Grzegórzecka Street, 31-531 Cracow, Poland; ^2^The Second Department of General Surgery, Faculty of Medicine, Jagiellonian University Medical College, 21 Kopernika Street, 31-501 Cracow, Poland; ^3^Department of Microbiology, Faculty of Medicine, Jagiellonian University Medical College, 18 Czysta Street, 31-121 Cracow, Poland; ^4^Department of Diagnostics, Chair of Clinical Biochemistry, Faculty of Medicine, Jagiellonian University Medical College, 15a Kopernika Street, 31-501 Cracow, Poland; ^5^Department of Pathomorphology, Faculty of Medicine, Jagiellonian University Medical College, 16 Grzegórzecka Street, 31-531 Cracow, Poland; ^6^Department of Internal Medicine II, Thuringia Clinic, Teaching Hospital of the University of Jena, Rainweg 68, 07318 Saalfeld, Germany

## Abstract

*Background*. Inflammatory bowel disease results from the dysregulation of immune response to environmental and microbial agents in genetically susceptible individuals. The aim of the present study was to examine the effect of rifaximin and/or Mutaflor (*Escherichia coli* Nissle 1917, EcN) administration on the healing of acetic acid-induced colitis.* Methods*. Colitis was induced in male Wistar rats by rectal enema with 3.5% acetic acid solution. Rifaximin (50 mg/kg/dose) and/or Mutaflor (10^9^ CFU/dose) were given intragastrically once a day. The severity of colitis was assessed at the 8th day after induction of inflammation.* Results*. Treatment with rifaximin significantly accelerated the healing of colonic damage. This effect was associated with significant reversion of the acetic acid-evoked decrease in mucosal blood flow and DNA synthesis. Moreover, administration of rifaximin significantly reduced concentration of proinflammatory TNF-*α* and activity of myeloperoxidase in colonic mucosa. Mutaflor given alone was without significant effect on activity of colitis. In contrast, Mutaflor given in combination with rifaximin significantly enhanced therapeutic effect of rifaximin. Moreover, Mutaflor led to settle of the colon by EcN and this effect was augmented by pretreatment with rifaximin.* Conclusion*. Rifaximin and Mutaflor exhibit synergic anti-inflammatory and therapeutic effect in acetic acid-induced colitis in rats.

## 1. Introduction

Genetic and environmental factors are involved in pathogenesis of inflammatory bowel disease (IBD). IBD results from the dysregulation of immune response to environmental and microbial agents in genetically susceptible individuals [1, 2]. Intestinal microflora plays a role in promoting and maintaining inflammatory process in this disease [3]. The intestinal flora contains various pathogens such as* Clostridium perfringens, Enterococcus*, Enterobacteriaceae, and* Bacteroides*. These bacteria are present in the large intestine of every healthy person in high concentrations, but, in the normal condition, they are separated from the colonic wall by an impenetrable mucus layer and are tolerated by the host. In patients with IBD, this separation is disturbed; bacteria adhere to the mucosa and invade epithelial cells with concomitant inflammatory response [4]. Moreover, the concentration of mucosal bacteria is higher in patients with IBD than in healthy persons and this concentration is proportional to the severity of the disease [4, 5].

Rifaximin is a locally acting antibacterial agent that is practically unabsorbed after oral administration (absorption less than 0.4%) and for this reason this medicine is risk-free of systemic side effects. Rifaximin exhibits a broad-spectrum activity against enteric Gram-positive and Gram-negative bacteria. Rifaximin has been found to be useful in the treatment of traveler's diarrhea and irritable bowel syndrome and in preventing of the stress-induced gut inflammation [6–9]. There are studies showing that rifaximin is effective in the treatment of IBD. Clinical trials have indicated that administration of 800 mg rifaximin twice daily for 12 weeks induces remission with few adverse events in patients with moderately active Crohn's disease (CD) [10], and remission previously obtained with standard treatment can be sustained in patients with moderately active CD by administration of rifaximin as well [11]. Rifaximin has been also found to be effective in the treatment of ulcerative colitis [12–14].

Living microorganisms that enter gastrointestinal tract and exert a beneficial effect on the host are called probiotics. There are studies showing the therapeutic effect of probiotics in the prevention or treatment of the gastrointestinal tract diseases [15, 16].

*Escherichia coli *Nissle 1917 (EcN) is a nonpathogenic strain of the Enterobacteriaceae family. It was originally isolated by a physician Alfred Nissle during the First World War on the Balkan Peninsula from the feces of a soldier, who in contrast to his comrades, did not develop enterocolitis [17]. Study performed by Altenhoefer et al. [18] has tested the interference of EcN with* Salmonella* invasion of human embryonic intestinal epithelial INT407 cells. Simultaneous administration of EcN 1917 and* Salmonella enterica* serovar Typhimurium strain C17 resulted in up to 70% reduction of* Salmonella* invasion efficiency. Furthermore, invasion of* Yersinia enterocolitica*,* Shigella flexneri*,* Legionella pneumophila*, and even* Listeria monocytogenes* was inhibited by EcN without affecting the viability of the invasive bacteria. There are also studies indicating the therapeutic effect of the EcN in patients with IBD. Maintaining remission in ulcerative colitis by the treatment with EcN has been shown to be as effective as treatment with “the gold standard” mesalazine [19–22]. On the other hand, there is only one clinical study showing beneficial effect of EcN in maintaining remission in patients with colonic Crohn's disease [23]. Application of EcN has reduced the risk for relapse and minimized the need for glucocorticoids.

The above observations suggest that rifaximin and Mutaflor can influence the course of IBD. The aim of the present study was to compare the effect of treatment with rifaximin and Mutaflor on the healing of acetic acid-induced colitis in rats. In addition, we investigated whether administration of the combination of rifaximin plus Mutaflor leads to any synergic interaction of their therapeutic effects in this model of IBD.

## 2. Materials and Methods

### 2.1. Animals and Treatment

Studies were performed on 64 male Wistar rats weighing 250–270 g and were conducted following the experimental protocol approved by the First Local Commission of Ethics for the Care and Use of Laboratory Animals in Cracow (Permit Number 2/2013 released on January 16, 2013). Animals were housed in cages in room temperature and a 12 h light-dark cycle. Rats were fasted with free access to water for 18 h before induction of colitis. Later food and tap water were available* ad libitum*.

Animals were randomly divided into eight equal experimental groups: (1) control rats without induction of colitis and treated intragastrically (i.g.) with saline; (2) rats without induction of colitis and treated i.g. with* Escherichia coli* Nissle 1917 (EcN); (3) rats without induction of colitis and treated i.g. with rifaximin; (4) rats without induction of colitis and treated i.g. with the combination of rifaximin plus EcN; (5) rats treated i.g. with saline after induction of colitis; (6) rats treated i.g. with EcN after induction of colitis; (7) rats treated i.g. with rifaximin after induction of colitis; and (8) rats treated i.g. with the combination of rifaximin plus EcN after induction of colitis.

Colitis was induced by a rectal enema with 1 mL of 3.5% (v/v) acetic acid aqueous solution in rats anesthetized with ketamine (50 mg/kg i.p., Bioketan, Vetoquinol Biowet, Gorzów Wielkopolski, Poland). Acetic acid solution was administered through a polyethylene catheter inserted into the rectum. There are different models of acetic acid-induced colitis and the tip of catheter can be positioned from 1.2  [24] to 8 cm [25] proximal to the anus verge. For this reason we have chosen an intermediate depth of catheter insertion, 4.5 cm from the anus. Rats without induction of colitis obtained rectal enema with an aqueous saline solution administered in the same manner as a solution of acetic acid in animals with induction of colitis.

Rifaximin (50 mg/kg/dose; Xifaxan, Norgine B.V., Amsterdam, Netherlands) and/or the probiotic strain* Escherichia coli* Nissle 1917 (approx. 10^9^ CFU/dose, Mutaflor; Ardeypharm GmbH, Herdecke, Germany) were given i.g. once a day for 7 days, starting at the day of colitis induction. Each dose of Mutaflor was given 2 h after treatment with rifaximin. In rats treated with saline, each dose of saline was given at the same time as in animals treated with Mutaflor. The last administration of saline, rifaximin, Mutaflor, or the combination of rifaximin plus Mutaflor was carried out 24 h before the end of experiment.

At the 8th day of study, animals were anesthetized and the research was terminated. This single observation period was chosen because the protocol of our research has been prepared in accordance with the policy of 3Rs (Replacement, Reduction and Refinement). Previous studies have shown that the potential therapeutic effects of factors being tested are clearly visible on the 8th day of study after the seven-day treatment [26].

### 2.2. Measurement of Colonic Blood Flow and Colonic Damage

Seven days after rectal enema with saline or induction of colitis, rats were anesthetized again with ketamine. After opening the abdominal cavity and exposure of the colon, the rate of colonic blood flow was measured using laser Doppler flowmeter (PeriFlux 4001 Master monitor, Perimed AB, Järfälla, Sweden), as described previously [27]. The measurement of mucosal blood flow was performed every time in five parts of the descending and sigmoid colon and the main value of five records was expressed as the percentage of the value obtained in the animals from the control group. After the measurement of colonic blood flow, anesthetized animals were euthanized by exsanguination from the abdominal aorta. Then, the area of mucosal damage was measured, using a computerized planimeter (Morphomat, Carl Zeiss, Berlin, Germany), as described previously [28].

### 2.3. Biochemical Analysis

After measurement of the area of mucosal damage, biopsy samples of colonic wall or colonic mucosa were taken for histological examination and determination of mucosal DNA synthesis (an index of mucosal cell vitality and proliferation), mucosal interleukin-1*β* (IL-1*β*) and Tumor Necrosis Factor-*α* (TNF-*α*) concentration, and mucosal activity of myeloperoxidase. DNA synthesis was determined by measurement of [^3^H]thymidine incorporation ([6-^3^H]-thymidine, 20–30 Ci/mmol, Institute for Research, Production and Application of Radioisotopes, Prague, Czech Republic) into mucosal DNA as described previously [29]. The incorporation of labeled thymidine into DNA was determined by counting 0.5 mL DNA-containing supernatant in a liquid scintillation system. DNA synthesis was expressed as tritium disintegrations per minute per microgram of DNA (dpm/*μ*g DNA).

Samples of the colonic mucosa, in which the concentration of IL-1*β* and TNF-*α* was measured, were homogenized in phosphate buffer at 4°C. Then the homogenate was centrifuged and the concentration of IL-1*β* and TNF-*α* was determined in the supernatant using the Rat IL-1*β* Platinum ELISA (Bender MedSystem GmbH, Vienna, Austria) or Rat TNF-*α* ELISA Kit (Koma Biotech, Seoul, South Korea), respectively. The concentration of IL-1*β* and TNF-*α* in the colonic mucosa was expressed in nanograms per 1 gram of tissue.

Biopsy samples for measurement of mucosal myeloperoxidase activity were homogenized in ice-cold potassium phosphate and, until the marking was done, stored at the temperature of −60°C. Marking myeloperoxidase activity was performed with the use of a modification of the method described by Bradley et al. [30]. Results were expressed in units per gram of tissue.

### 2.4. Histological Examination of the Colon

Samples of the colon were fixed in 10% buffered formaldehyde and embedded in paraffin. Paraffin sections were stained with hematoxylin and eosin and examined by the pathologist uninformed about treatment given. The histological grading of colonic damage such as ulceration, inflammation, depth of the lesion, and fibrosis was determined using a scale of Vilaseca et al. [31] as described in detail previously [32].

### 2.5. Microbiological Analysis

Feces samples were taken and transported in deep-freeze conditions to the microbiological laboratory where DNA was extracted according to the previously described procedure [33].

*Escherichia coli *and* Enterococcus* species in the fecal samples were quantified by quantitative real time PCR (qPCR) according to the method described by Pilarczyk-Zurek et al. [34] and Ryu et al. [35], respectively. To detect specific DNA sequences, ready-to-use JumpStart Taq ReadyMix kit (Sigma-Aldrich, Saint Louis, USA), fluorescently FAM dye labeled probe (F) GGGAGTAAAGTTAATACCTTTGC, (R) CTCAAGCTTGCCAGTATCAG, FAM-CGCGATCACTCCGTGCCAGCAGCCGCGGATCGCG-BHQ1 (GenoMed) for* E. coli*, and (ECST748F) AGAAATTCCAAACGAACTTG and (ENC854R) CAGTGCTCTACCTCCATCATT for* Enterococcus* spp. (SYBR Green dye) were used. A standard curve was prepared. DNA from given numbers of* E. coli *ATCC25922 and separately* E. faecalis* ATCC19433 was added in serial dilutions from 10^1^ to 10^7^ cells to a series of qPCRs. The reactions were carried out in a CFX96 thermocycler (BioRad). Detection and quantitation were linear over the range of DNA concentrations examined. To determine the number of both bacterial species cells, the fluorescent signals detected from DNA feces samples (in duplicate) in the linear range of the assay were averaged and compared to a standard curve.

EcN DNA in the fecal samples were detected by triplex PCR method described by Blum-Oehler et al. [36] using three primers pairs: Muta 5/6; Muta 7/8; and Muta 9/10. The reactions were carried out on the CFX96 thermocycler (BioRad). All PCR products were analyzed by electrophoresis through 3% agarose gels (Prona).

### 2.6. Statistical Analysis

Statistical analysis of the data was carried out by one-way analysis of variance (ANOVA) followed by Tukey's multiple comparison test using GraphPad Prism (GraphPad Software, San Diego, CA, USA). Differences were considered to be statistically significant when *P* was less than 0.05.

## 3. Results

**Figure 1:**
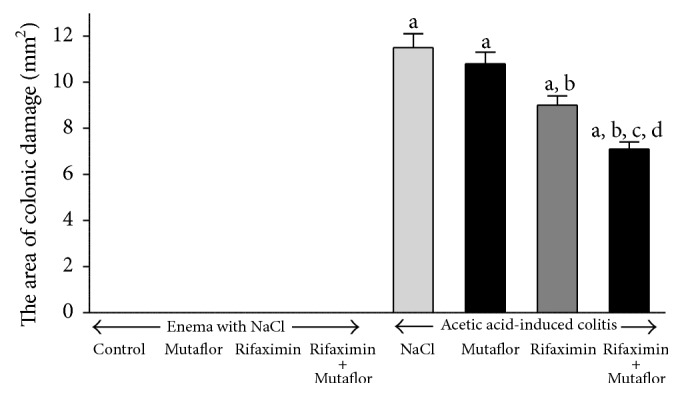
Influence of* E. coli* Nissle 1917 (Mutaflor) and rifaximin on the area of colonic lesions in rats without or with acetic acid-induced colitis. Mean value ± SEM. *N* = 8 animals in each experimental group. ^a^ *P* < 0.05 compared to control saline-treated rats without induction of colitis; ^b^ *P* < 0.05 compared to colitis + NaCl; ^c^ *P* < 0.05 compared to colitis + Mutaflor; and ^d^ *P* < 0.05 compared to colitis + rifaximin.

**Figure 2:**
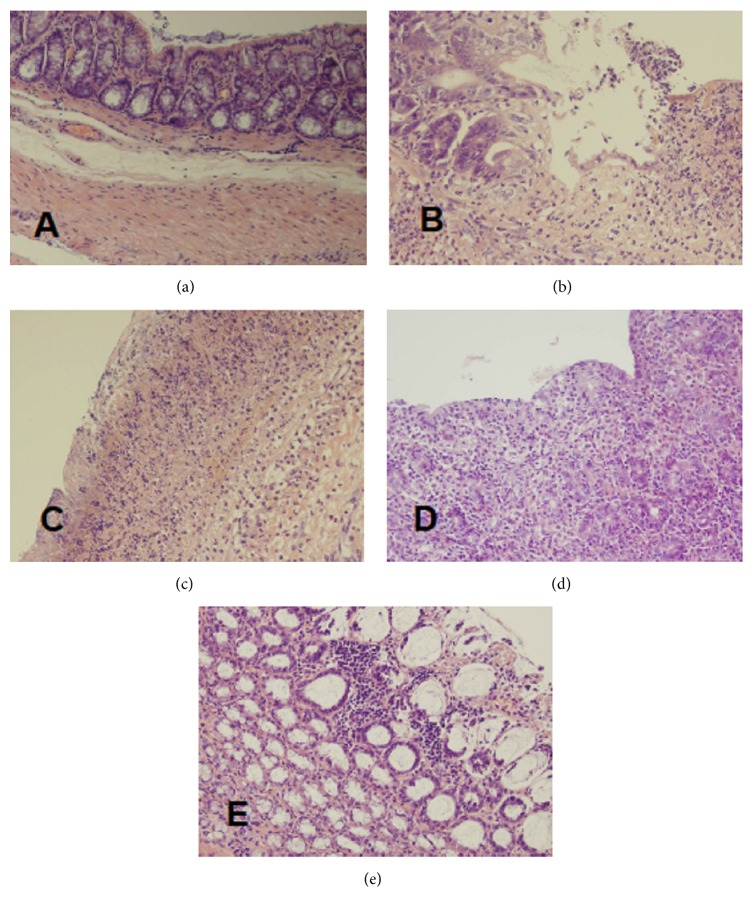
Histological features of the rat colonic mucosa stained by haematoxylin and eosin (original magnification 400x). (a) Control rats without induction of colitis and treated with saline for 7 days; (b) rats with colitis treated with saline for 7 days; (c) rats with colitis treated with Mutaflor for 7 days; (d) rats with colitis treated with rifaximin for 7 days; and (e) rats with colitis treated with the combination of rifaximin plus Mutaflor for 7 days.

Macroscopic and microscopic evaluation of the colon showed no damage in control saline-treated animals without induction of colitis (Figure 1, Table 1, and Figure 2(a)). No colonic damage was also seen in animals without induction of colitis and treated with Mutaflor, rifaximin, or a combination thereof (Figure 1, Table 1). Rectal enema with acetic acid solution caused induction of colitis in all rats subjected to this procedure. In saline-treated rats, 7 days after the induction of colitis, the area of mucosal damage reached a value of 11.5 ± 0.6 mm^2^. Microscopic examination of the colon showed the presence of large lesions reaching the level of muscular membrane or even serous membrane (Table 1, Figure 2(b)). This alteration was associated with moderate or heavy inflammatory infiltration and the presence of mild fibrosis.

Macroscopic examination showed that treatment with Mutaflor given alone after induction of colitis tended to reduce the area of mucosal damage in the colon; however this effect was statistically insignificant. Microscopic examination of the colon showed no effect of Mutaflor given alone on a size of damage and inflammatory infiltration in rats with colitis. Only a depth of colonic damage and a grade of fibrosis were slightly reduced in some animals (Table 1, Figure 2(c)).

In contrast, treatment with rifaximin given alone significantly reduced the area of mucosal damage by 22% when compared to animals with colitis treated with saline (Figure 1). Microscopic examination showed that the administration of rifaximin reduces the extent of colonic damage, inflammatory infiltration, and development of fibrosis (Table 1, Figure 2(d)).

Maximal reduction of the area of mucosal damage in macroscopic examination was observed in rats with colitis treated with the combination of rifaximin plus Mutaflor. The area of damage in those group of rats was significantly lower than in animals treated with saline, rifaximin, or Mutaflor given alone (Figure 1). Also microscopic evaluation showed that treatment with the combination of rifaximin plus Mutaflor maximally reduced the colonic damage in rats with colitis (Table 1, Figure 2(e)).

**Figure 3:**
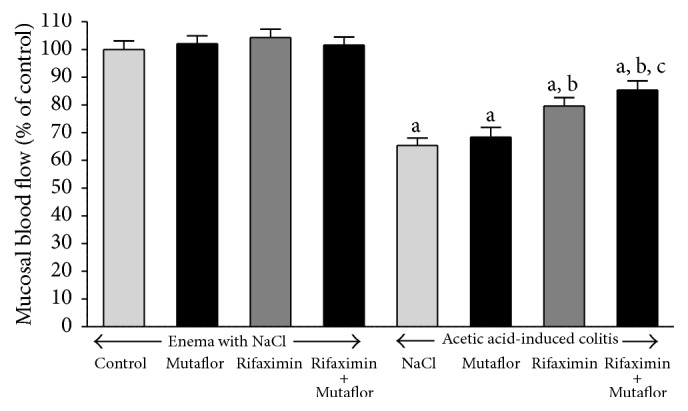
Influence of* E. coli* Nissle 1917 (Mutaflor) and rifaximin on mucosal blood flow in the colon in rats without or with acetic acid-induced colitis. Mean value ± SEM. *N* = 8 animals in each experimental group. ^a^ *P* < 0.05 compared to control saline-treated rats without induction of colitis; ^b^ *P* < 0.05 compared to colitis + NaCl; and ^c^ *P* < 0.05 compared to colitis + Mutaflor.

In the rats without induction of colitis, administration of Mutaflor and rifaximin given alone or in their combination failed to affect mucosal blood flow in the colon (Figure 3). Induction of colitis significantly reduced blood flow in colonic mucosa by around 35% in comparison to a value observed in control saline-treated rats. In rats with colitis, administration of Mutaflor given alone tended to improve colonic blood flow, but this effect was statistically insignificant. In contrast, the administration of rifaximin significantly improved blood flow through the colonic mucosa. The greatest improvement in blood flow in colonic mucosa of rats with colitis was observed after treatment with the combination of rifaximin plus Mutaflor (Figure 3).

**Figure 4:**
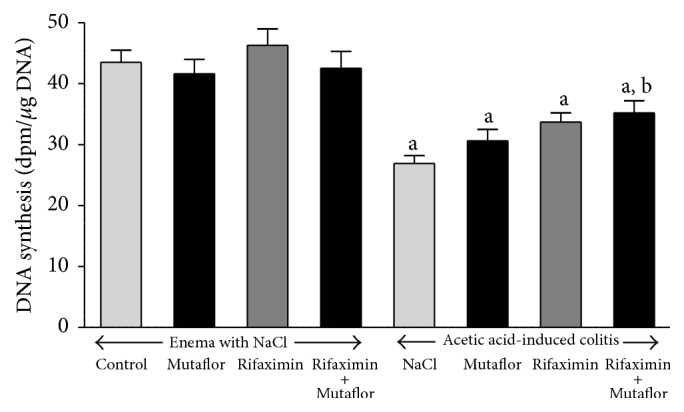
Influence of* E. coli* Nissle 1917 (Mutaflor) and rifaximin on DNA synthesis in colonic mucosa in rats without or with acetic acid-induced colitis. Mean value ± SEM. *N* = 8 animals in each experimental group. ^a^ *P* < 0.05 compared to control saline-treated rats without induction of colitis; ^b^ *P* < 0.05 compared to colitis + NaCl.

In rats without induction of colitis, administration of Mutaflor or rifaximin given alone or in their combination was without any effect on DNA synthesis in colonic mucosa (Figure 4). Induction of colitis by the enema with acetic acid led to reduction in DNA synthesis in colonic mucosa by 38%. Administration of Mutaflor or rifaximin given alone did not significantly affect DNA synthesis in colonic mucosa in rats with colitis. Treatment with the combination of rifaximin plus Mutaflor partly, but significantly reversed the colitis-evoked reduction in mucosal DNA synthesis in the colon (Figure 4).

**Figure 5:**
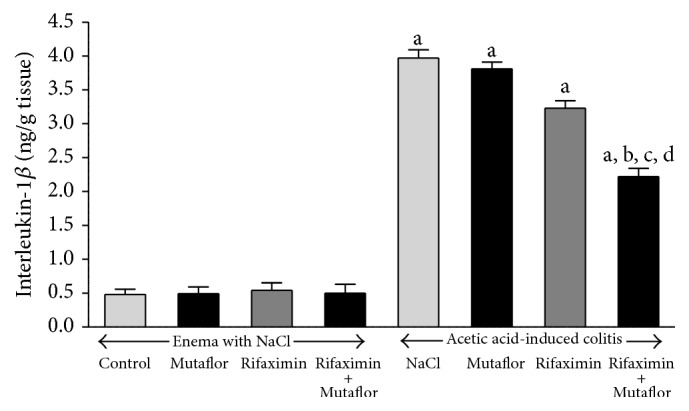
Influence of* E. coli* Nissle 1917 (Mutaflor) and rifaximin on of interleukin-1*β* concentration in colonic mucosa in rats without or with acetic acid-induced colitis. Mean value ± SEM. *N* = 8 animals in each experimental group. ^a^ *P* < 0.05 compared to control saline-treated rats without induction of colitis; ^b^ *P* < 0.05 compared to colitis + NaCl; ^c^ *P* < 0.05 compared to colitis + Mutaflor; and ^d^ *P* < 0.05 compared to colitis + rifaximin.

**Figure 6:**
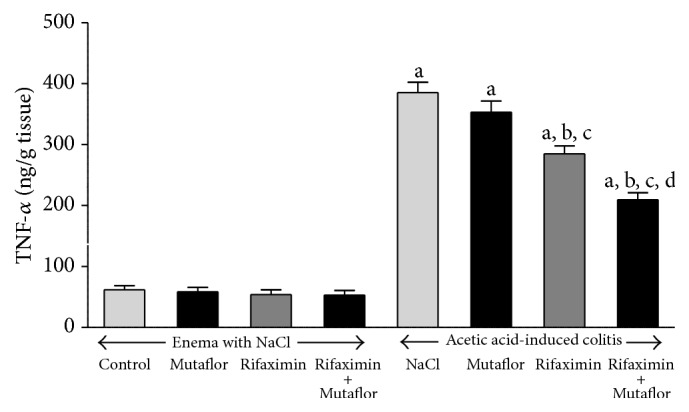
Influence of* E. coli* Nissle 1917 (Mutaflor) and rifaximin on of Tumor Necrosis Factor-*α* concentration in colonic mucosa in rats without or with acetic acid-induced colitis. Mean value ± SEM. *N* = 8 animals in each experimental group. ^a^ *P* < 0.05 compared to control saline-treated rats without induction of colitis; ^b^ *P* < 0.05 compared to colitis + NaCl; ^c^ *P* < 0.05 compared to colitis + Mutaflor; and ^d^ *P* < 0.05 compared to colitis + rifaximin.

In rats without induction of colitis, intragastric administration of Mutaflor or rifaximin for 7 days failed to affect mucosal concentration of interleukin-1*β* (IL-1*β*) or Tumor Necrosis Factor-*α* (TNF-*α*) in the colon (Figures 5 and 6, resp.). Induction of colitis caused more than 8-fold increase in concentration of IL-1*β* and more than 6-fold increase in concentration of TNF-*α* in colonic mucosa. Administration of Mutaflor after induction of colitis was without significant effect on mucosal concentration of IL-1*β* or TNF-*α* in the colon. Rifaximin administered alone caused a partial decrease in the level of IL-1*β* and TNF-*α* in colonic mucosa of animals with colitis. However, only the reduction of TNF-*α* concentration was statistically significant when compared to level observed in rats with colitis treated with saline. Maximal reduction in concentration of IL-1*β* and TNF-*α* in colonic mucosa was observed after administration of combination of rifaximin plus Mutaflor (Figures 5 and 6).

**Figure 7:**
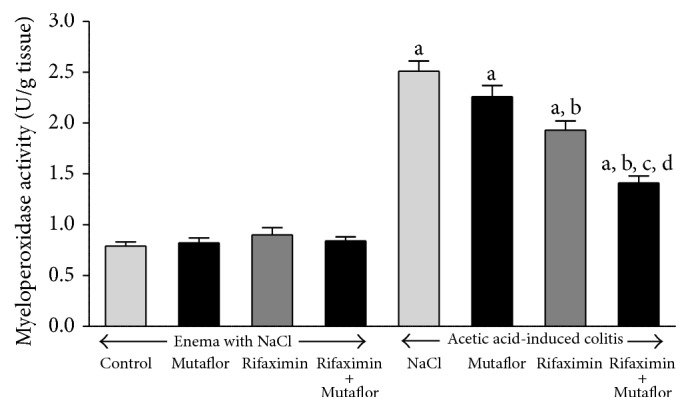
Influence of* E. coli* Nissle 1917 (Mutaflor) and rifaximin on myeloperoxidase activity in colonic mucosa in rats without or with acetic acid-induced colitis. Mean value ± SEM. *N* = 8 animals in each experimental group and each time of observation. ^a^ *P* < 0.05 compared to control saline-treated rats without induction of colitis; ^b^ *P* < 0.05 compared to colitis + NaCl; ^c^ *P* < 0.05 compared to colitis + Mutaflor; and ^d^ *P* < 0.05 compared to colitis + rifaximin.

Administration of Mutaflor or rifaximin given alone as well as treatment with the combination of rifaximin plus Mutaflor was without any significant effect on mucosal myeloperoxidase activity in the colon in the rats without colitis induction (Figure 7). Induction of colitis caused more than a 3-fold increase in myeloperoxidase activity in colonic mucosa. Treatment with Mutaflor tended to reduce the colitis-evoked increase in myeloperoxidase activity in the colonic mucosa, but this effect was statistically insignificant. In contrast, administration of rifaximin resulted in a significant reduction in mucosal activity of myeloperoxidase in rats with colitis. Maximal reduction of the colitis-induced increase in mucosal myeloperoxidase activity as observed after treatment with combination of rifaximin and Mutaflor (Figure 7).

In control rats without induction of colitis and treated i.g. with saline, the concentration of* E. coli* was 2.41 × 10^5^ colony forming units (CFU) per gram of feces (Table 2). In rats without induction of colitis, treatment with Mutaflor tended to increase the concentration of* E. coli* in feces, whereas administration of rifaximin tended to reduce the number of those bacteria in the stool. However, both of those results were statistically insignificant. Induction of colitis caused statistically significant increase in concentration of* E. coli* in the stool. This effect of colitis on the number of* E. coli* in feces was significantly reversed by treatment with Mutaflor and rifaximin given alone or in their combination (Table 2).

In rats without induction of colitis and treated with saline, the concentration of* Enterococcus *spp. was 3.54 × 10^7^ colony forming units (CFU) per gram of feces (Table 3). In rats without induction of colitis, treatment with Mutaflor or rifaximin given alone or in their combination significantly reduced the concentration of* E. *spp. in feces. Induction of colitis significantly increased the concentration of* E. *spp. in the stool. Treatment with Mutaflor and rifaximin given alone or in their combination significantly reversed the colitis-evoked increase in the number of* E. *spp. in feces (Table 3).

**Figure 8:**
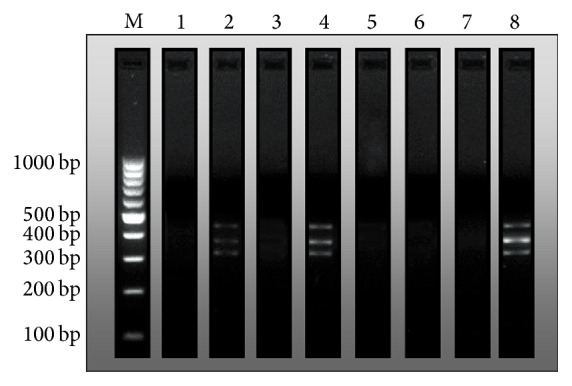
Determination of Muta 5/6 (361 bp), Muta 7/8 (427 bp), and Muta 9/10 (313 bp) amplicons by PCR method as indices of the presence of* E*.* coli* Nissle 1917 (EcN). Representative findings in (line 1) control rats without induction of colitis and treated intragastrically (i.g.) with saline; (line 2) rats without induction of colitis and treated i.g. with Mutaflor; (line 3) rats without induction of colitis and treated i.g. with rifaximin; (line 4) rats without induction of colitis and treated i.g. with the combination of rifaximin plus Mutaflor; (line 5) rats treated i.g. with saline after induction of colitis; (line 6) rats treated i.g. with Mutaflor after induction of colitis; (line 7) rats treated i.g. with rifaximin after induction of colitis; and (line 8) rats treated i.g. with the combination of rifaximin plus Mutaflor after induction of colitis. Line M—DNA mass ruler.

In all rats without treatment with Mutaflor, the presence of* E. coli* Nissle 1917 (EcN) was not detected (Figure 8). Administration of Mutaflor resulted in colonization of the large intestine by EcN in all rats without induction of colitis. This effect was increased in animals pretreated with rifaximin before Mutaflor administration. In animals with colitis, the colonization of the large intestine by EcN following administration of Mutaflor was less effective. Administration of Mutaflor given alone in rats with colitis led to the presence of primers specific to the EcN only in two cases on 8 observations. The addition of rifaximin before each dose of Mutaflor improved colonization of the colon by EcN. The presence of the EcN was observed in all animals in this experimental group (Figure 8).

## 4. Discussion

Our present study has shown that treatment with rifaximin accelerates the healing of acetic acid-induced colitis. This effect has been found as a faster reduction in the area of colonic damage, as well as a decrease in mucosal level of myeloperoxidase (MPO), interleukin-1*β* (IL-1*β*), and Tumor Necrosis Factor-*α* (TNF-*α*).

MPO is an enzyme most abundantly present in azurophilic granules of neutrophil granulocytes. Antimicrobial function of neutrophils is related, among others, to activity of MPO and possibility to generate hypochlorous acids and reactive oxygen species (ROS) during the respiratory burst [37]. MPO is released by activated neutrophils and for this reason tissue activity of MPO reflects the degree of tissue infiltration by neutrophils and may be used as an indirect marker of tissue oxidative stress [37, 38]. In turn, IL-1*β* and TNF-*α* are important proinflammatory cytokines. IL-1*β* is a proinflammatory cytokine responsible for initiating the release of a cascade of proinflammatory factors during inflammation [39]. Administration of rifaximin after induction of colitis has decreased the activity of MPO and reduced concentration of IL-1*β* and TNF-*α* in colonic mucosa, reflecting the reduction in the local inflammatory reaction.

Treatment with rifaximin has also affected blood flow and DNA synthesis in colonic mucosa in rats with acetic acid-induced colitis. Induction of colitis has strongly reduced those parameters, whereas administration of rifaximin has significantly improved blood flow and DNA synthesis in rats with colitis. Mucosal blood flow plays an important role in the protection and healing of mucosa in the gut [40, 41]. Previous studies have shown that exposure of gastric mucosa to potentially noxious factors results in little or no damage, as long as adequate blood flow is maintained, whereas reduction in mucosal blood flow leads to severe gastric injury [41].

Rate of mucosal DNA synthesis can be recognized as an index of cell vitality and cell proliferation. Previous studies have shown that inhibition of mucosal cell proliferation or excessive apoptosis results in the development of ulcers [42, 43], whereas a stimulation of mucosal cell proliferation exhibits protective and healing in the gastrointestinal tract [28, 44–46].

Therapeutic effect of rifaximin in acetic acid-induced colitis shown in our present study is in harmony with previous experimental [47] and clinical studies [10–13].

In contrast to effects obtained after treatment with rifaximin, we have found that administration of Mutaflor tends to improve the healing of acetic acid-induced colitis, but this effect is weak and statistically insignificant. Moreover, the presence of* Escherichia coli* strain Nissle 1917 (EcN) has been found only in feces of two per eight rats treated with Mutaflor alone after induction of colitis. This observation explains why studies showing the therapeutic effect of EcN in colitis are so few and far between. There are some experimental studies performed on rodents showing preventive and/or therapeutic effect of pretreatment or treatment with EcN in colitis evoked by dextran sodium sulfate [48], trinitrobenzene sulfonic acid [49], or transfer of CD4^+^ CD62L^+^ T lymphocytes from BALB/c mice in SCID mice [48]. Moreover, there are three clinical studies performed in adult patients [19–21] and one in children and adolescents [50] showing that EcN (Mutaflor) given orally is useful in preventing relapses in inactive ulcerative colitis (UC) and its efficacy is comparable to effects of standard therapy with mesalazine. Moreover, studies performed by Rembacken et al. [21] have found that oral treatment with EcN leads to remission in the similar percentage of patients with active ulcerative colitis as treatment with mesalazine. Unfortunately, their data are difficult to interpret because all patients at the same time were treated with hydrocortisone acetate enemas or prednisone given orally according to the severity of disease. Moreover, all patients received a 1-week course of oral gentamicin [21].

There is also clinical trial examining the potential therapeutic effect EcN in UC [22]. Patients with moderate distal activity in UC were assigned to treatment with either 40, 20, or 10 mL enemas containing 10^8^ EcN/mL or placebo. Authors have found that according to an intent-to-treat-population analysis the number of responders was not significantly higher in EcN group than in the placebo group. On the other hand, they have also reported that the Jonckheere-Terpstra rank correlation for dose-dependent efficiency indicated a significant correlation of per-protocol responder rates. Time to remission was shortest in patients treated with EcN 40 mL. However, it must be pointed out that groups of patient were not equivalent.

In the case of Crohn's disease (CD), there is only one clinical study showing that administration of EcN can help in maintaining remission in this disease [23].

The most important finding of our present study is the observation that administration of combination of rifaximin plus EcN in the course of acetic acid-induced colitis generates a greater therapeutic effect than any of these agents given alone. It was manifested by a statistically significant acceleration of healing of colonic damage, as well as by a reduction in local inflammatory process found as a decrease in MPO activity and a reduction in concentration of IL-1*β* and TNF-*α* in colonic mucosa. Moreover, we have found that treatment with combination of rifaximin plus EcN maximally improves blood flow and DNA synthesis in mucosa of the colon in rats with colitis.

Another important finding of our present study was the observation that pretreatment with rifaximin before administration of EcN favors the colonization of the colon by EcN. The presence of EcN has been found in all rats treated with the combination of rifaximin plus Mutaflor. Currently, great attention is given to the role of commensal bacteria in the pathogenesis of IBD. There are studies showing an increase in colonic population of Enterobacteriaceae, including* E. coli* in patients with UC and CD versus a control group [51]. Kleessen et al. [51] have found a bacterial invasion of mucosa in colonic specimens of UC patients, as well as in ileal and colonic specimens obtained from CD patients. In contrast to that, no bacteria were detected in tissues of healthy humans [51]. Similar findings have been found by Mylonaki et al. [52]. They have detected higher number of epithelium-associated* E. coli* in active than inactive UC or controls. Epithelium-associated* E. coli* counts were also higher in CD. Moreover* E. coli* were also found as individual bacteria and in clusters in the lamina propria in UC and CD patients but in none of the controls.

In harmony with the above-mentioned observations are recent studies performed by Elliott et al. [53] and Vazeille et al. [54]. Elliott et al. [53] have found that intramacrophage* E. coli* are commonly observed in lamina propria macrophages in mucosal biopsies from CD patients, rarely in UC and not at all in healthy controls. Authors have concluded that persistence of* E. coli* within macrophages located in lamina propria may provide a stimulus for chronic inflammation. The role of* E. coli* in the pathogenesis of IBD has also been confirmed by findings of Vazeille et al. [54]. They have found that monocyte-derived macrophages from CD patients are impaired in the ability to control intracellular adherent-invasive* E. coli* and exhibit disordered cytokine profile. Moreover, currently performed meta-analysis has revealed that intestinal colonization with phylogenetic group B2* E. coli* is associated with UC [55].

Data mentioned above and our results taken together suggest that substitution of other, potentially pathogenic strains of* E. coli* by nonpathogenic strain Nissle 1917 plays an important role in therapeutic effect of coadministration of rifaximin plus Mutaflor in acetic acid-induced colitis. Substitution of other* E. coli* strains by EcN is also important due to the rapid development of resistance of* E. coli* against rifaximin in the case of use of this antibiotic [56]. Moreover, our present study has shown treatment with Mutaflor and rifaximin given alone or in their combination significantly reversed the colitis-evoked increase in the colonic number of* Enterococcus *spp.

Finally, we conclude that rifaximin and Mutaflor exhibit synergic anti-inflammatory and therapeutic effect in acetic acid-induced colitis in rats. This observation suggests that rifaximin plus Mutaflor may be the optimal choice in the treatment of colitis by probiotics.

## Figures and Tables

**Table 1:** Influence of *Escherichia coli *Nissle 1917 (Mutaflor) and rifaximin on morphological signs of colonic damage in rats without or with acetic acid-induced colitis observed 8 days after rectal administration of saline or acetic acid solution (colitis).

	Morphological changes			
	Grading of colonic damage (0–2)	Inflammatory infiltration (0–3)	Depth of damage (0–3)	Fibrosis (0–3)
Saline (control)	0	0	0	0
Mutaflor	0	0	0	0
Rifaximin	0	0	0	0
Rifaximin + Mutaflor	0	0	0	0
Colitis + NaCl	3	2-3	2	1-2
Colitis + Mutaflor	3	2-3	1-2	1
Colitis + rifaximin	2-3	1-2	1-2	1
Colitis + rifaximin + Mutaflor	2	1	1	0-1

Numbers represent the predominant histological grading in each group.

**Table 2:** Influence of treatment with *Escherichia coli *Nissle 1917 (Mutaflor) and/or rifaximin and induction of colitis on the total number of *Escherichia coli* identified by qPCR in feces samples.

Experimental groups	The number of bacteria (CFU/g)
Control	2.41 × 10^5^ ± 7.59 × 10^4^
Mutaflor	6.42 × 10^6^ ± 3.21 × 10^6^
Rifaximin	2.95 × 10^2^ ± 1.69 × 10^2^
Rifaximin + Mutaflor	5.54 × 10^4^ ± 2.28 × 10^4^
Colitis + NaCl	7.57 × 10^8^ ± 4.38 × 10^8^ ^a^
Colitis + Mutaflor	1.11 × 10^7^ ± 4.22 × 10^6^ ^b^
Colitis + rifaximin	1.27 × 10^3^ ± 6.68 × 10^2^ ^b^
Colitis + rifaximin + Mutaflor	4.52 × 10^5^ ± 1.68 × 10^5^ ^b^

Mean value ± SEM. *N* = 8 observations in each experimental group. ^a^ *P* < 0.05 compared to control; ^b^ *P* < 0.05 compared to colitis + NaCl.

**Table 3:** Influence of Mutaflor (*Escherichia coli *Nissle 1917), rifaximin, and colitis applied alone or in their combination on the number of *Enterococcus *spp. identified by qPCR in feces samples.

Experimental groups	The number of bacteria (CFU/g)
Control	3.54 × 10^7^ ± 5.90 × 10^4^
Mutaflor	3.23 × 10^5^ ± 5.73 × 10^3^ ^a^
Rifaximin	6.88 × 10^1^ ± 3.78 × 10^1^ ^a^
Rifaximin + Mutaflor	3.59 × 10^2^ ± 7.69 × 10^1^ ^a^
Colitis + NaCl	4.34 × 10^8^ ± 5.68 × 10^6^ ^a^
Colitis + Mutaflor	2.45 × 10^7^ ± 3.22 × 10^5^ ^a,b^
Colitis + rifaximin	1.45 × 10^2^ ± 7.26 × 10^1^ ^a,b,c^
Colitis + rifaximin + Mutaflor	6.34 × 10^4^ ± 7.05 × 10^3^ ^a,b,c^

Mean value ± SEM. *N* = 8 observations in each experimental group. ^a^ *P* < 0.05 compared to control; ^b^ *P* < 0.05 compared to colitis + NaCl; and ^c^ *P* < 0.05 compared to colitis + Mutaflor.
